# A Novel Eye Drop Candidate for Age-Related Macular Degeneration Treatment: Studies on its Pharmacokinetics and Distribution in Rats and Rabbits

**DOI:** 10.3390/molecules25030663

**Published:** 2020-02-04

**Authors:** Eun-Jeong Choi, Go-Wun Choi, Ju Hee Kim, Hee-Woon Jang, Ju-Hee Lee, Hyun Ju Bae, Young Gwan Kim, Yong-Bok Lee, Hea-Young Cho

**Affiliations:** 1 College of Pharmacy, CHA University, 335 Pangyo-ro, Bundang-gu, Seongnam-si, Gyeonggi-do 13488, Korea; choiej5048@gmail.com (E.-J.C.); gwchoi153@gmail.com (G.-W.C.); 20135107@ppharm.org (J.H.K.); heewoon21@gmail.com (H.-W.J.); 2 Kukje Pharma R&D Center, Sanseong-ro 47, Ansan, Gyeonggi-do 15438, Korea; jhlee@kukjepharm.co.kr (J.-H.L.); hjbae1201@gmail.com (H.J.B.); ykkim@kukjepharm.co.kr (Y.G.K.); 3 College of Pharmacy, Chonnam National University, 77 Yongbong-ro, Buk-Gu, Gwangju 61186, Korea; leeyb@chonnam.ac.kr

**Keywords:** CK41016, dry age-related macular generation, pharmacokinetics, tissue distribution, Caco-2

## Abstract

Age-related macular degeneration (AMD) is wearing down of macula of retina, causing a blur or loss of vision in the center of the visual field. It can be categorized into dry or wet AMD. Until now, medical treatments for dry AMD have not been developed yet. The aim of this study was to evaluate pharmacokinetics (PKs) and tissue distribution of CK41016, a novel candidate for dry AMD, after intravenous (IV) or eye drop administration in rats and rabbits. In addition, a simple and sensitive bioanalytical method for CK41016 using ultra performance liquid chromatography-tandem mass spectrometer (UPLC-MS/MS) was developed. PK parameters were estimated by compartmental analysis using a WinNonlin^®^ software version 8.1 (a Certara™ company). A PK model of CK41016 was well-described by the two-compartment model. The tissue-to-plasma partition coefficient (Kp) of CK41016 was the highest in the vitreous humor of rats and the cornea of rabbits after eye drop administration. In addition, the Caco-2 cell transporter assay confirmed that CK41016 was not an active substrate for the efflux transporter. In summary, the PKs and tissue distribution of CK41016 were successfully evaluated and investigated whether this drug was a substrate of efflux transporters.

## 1. Introduction

Age-related macular degeneration (AMD) is one of the causes of adult blindness, due to macular damage of the retina. Symptoms of this disease include distorted vision, blurred vision, a loss in contrast sensitivity, slow recovery of visual function after exposure to bright light, and no vision in the center of the visual field [1].

AMD can be classified into wet AMD and dry AMD. About 10% of patients with AMD have wet AMD with choroidal neovascularization (CNV), which can cause vision loss. However, the development of laser coagulation, anti-VEGF medication, and photodynamic therapy has reduced the chances of vision loss for patients with wet AMD [2,3]. On the other hand, about 90% of patients with AMD have dry AMD, which is associated with the formation of drusen, containing lipids, proteins, and undigested cell wastes. Drusen can result from stressors such as oxidative stress caused by smoking and aging, and they are located between the retinal pigment epithelium (RPE) and Bruch’s membrane [4]. In the late stage of the disease progression, dry AMD can often be a leading step into geographic atrophy (GA) or wet AMD.

Several methods have been studied to treat dry AMD, including physical treatment, cell-based therapy, and drugs. Physical treatments, such as lasers [5,6,7], implantable telescopic lenses [8,9], and rheopheresis [10,11] have shown no significant benefits in clinical trials. Cell-based therapy [12,13] is the most potent therapy for recovering damaged RPE layers using stem cells. In the case of drugs, a few drug candidates are in the development process, with phase 2 or 3 clinical trials [14,15,16,17]. Drugs with the following mechanisms of action are undergoing clinical trials: preventing accumulation of drusen, anti-inflammation, increasing choroidal blood flow, and inhibiting the death of RPE cells influenced by drusen. The administration routes of drugs for dry AMD are mostly oral administration and intraocular injection [18], with a few reports on eye drop formulation [15,19].

CK41016 is a new drug candidate as an eye drop for treating dry AMD. Up to now, there are two reported eye drop formulations of dry AMD drugs in clinical trials, including OT-551 with antioxidant action and MC-1101 increasing blood flow. However, neither of the candidates is appropriate for treating the late stage of dry AMD. On the other hand, CK41016 is suitable for treating late-stage dry AMD, because it can inhibit the death of RPE cells [20].

In the case of topical administrations for dry AMD, it is critical that drugs be delivered to the back of the eye. However, it is hard to reach the back of the eyes through eye drop formulation because of many barriers in the eyes [21]. Therefore, it is necessary to confirm whether a drug is distributed to the back of the eyes or not.

To better understand the pharmacokinetics (PKs) and tissue distribution of CK41016, an efflux transport assay was conducted to investigate its absorption and distribution mechanism. P-glycoprotein (P-gp), breast cancer resistance protein (BCRP), and multidrug resistance protein (MRP) are the three major efflux transporters that affect a drug’s absorption and kinetics, drug-drug interactions, and its safety profiles [22]. The efflux transporters, such as P-gp, are expressed in the human RPE [23,24], playing a role in limiting the bioavailability of a drug in the eye [25]. Caco-2 cells that express efflux transporters are usually used as a standard screening tool to investigate the absorption and distribution mechanisms of drug candidates. Therefore, a Caco-2 monolayer cell model was selected for the efflux transport assay.

In this study, PK parameters and tissue distributions of CK41016 were evaluated after IV or eye drop administration in rats and rabbits. In addition, the efflux transport of CK41016 was investigated using Caco-2 cells.

## 2. Results and Discussion

### 2.1. Determination of CK41016 Using Ultra Performance Liquid Chromatography-Tandem Mass Spectrometer (UPLC-MS/MS)

**Figure 1:**
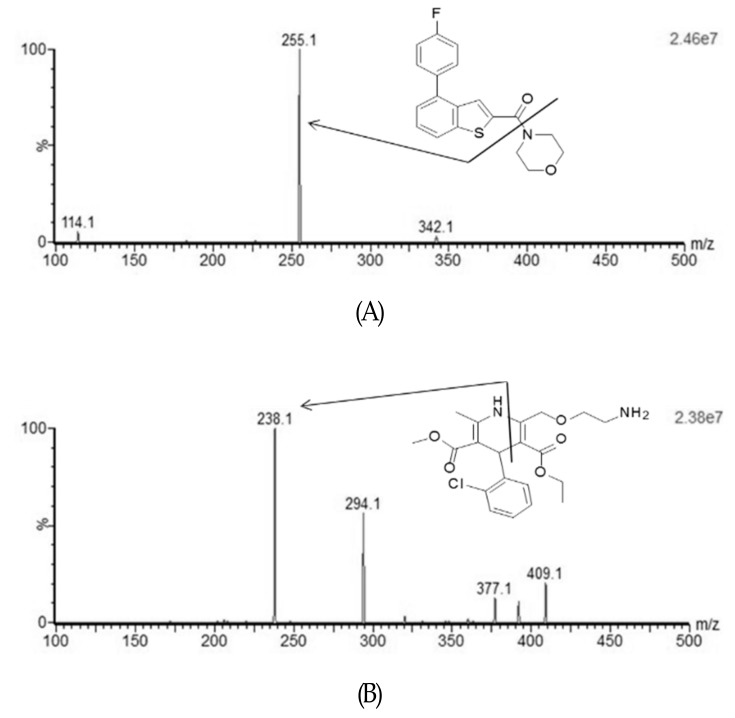
Positive ion electrospray mass scan spectra of CK41016 (**A**) and the internal standard (IS) (**B**).

In this study, an improved sensitive and selective UPLC-MS/MS method was developed for determining the analyte in biological samples. Chromatographic condition, sample preparation method, and mass spectrometric parameters, such as capillary voltage, collision energy, desolvation temperature, ion source temperature, and flow rates of desolvation and cone gases were optimized to determine CK41016 and the internal standard (amlodipine, IS). Full-scan product mass spectra of CK41016 and IS are shown in Figure 1. Multiple reaction monitoring (MRM) transitions for CK41016 and IS were at *m/z* 342.1→255.1 and 409.1→238.1, respectively. For CK41016, the daughter ion could be detected at *m/z* 255.1 and 144.1. We used *m/z* 255.1 for the daughter ion due to its high intensity. Various collision energies were examined to obtain the best abundance of peak. When 20 eV of collision energy was used, the highest sensitivity of CK41016 was obtained. Therefore, 20 eV was chosen as the collision energy for CK41016.

Considering the selectivity and effects of the coeluting peak for CK41016, we tried various mobile phases, such as water, acidic, and a buffer solution to optimize the liquid chromatography method. When 0.1% formic acid in water and 0.1% formic acid in acetonitrile were used, the intensity was higher than in other conditions. In addition, tetrahydrofuran was added to mobile phase A and B to avoid interference with detection of CK41016. To achieve stable base line and better peak shape, we also tested various columns with different sizes and compounds bonded to silica, including HALO C_18_ column (50 mm × 2.1 mm, 2 μm particle size, Advanced Materials Technology, Wilmington, DE, USA); Kinetex C_18_ column (50 mm × 2.1 mm, 1.7 μm particle size, Phenomenex, Torrance, CA, USA); Capcell core C_18_ column (50 mm × 2.1 mm, 2.7 μm particle size, Shiseido, Osaka, Japan); Unison UK-C_18_ column (50 mm × 2.0 mm, 3 μm particle size, Imtakt Corp., Tokyo, Japan); and Kinetex biphenyl column (2.1 mm × 50 mm, 1.7 µm particle size, Phenomenex, Torrance, CA, USA). Kinetex biphenyl column exhibited the best sensitivity, selectivity, symmetric peak shape, and intensity. The retention time was 2.35 min for CK41016 and 1.18 min for IS.

For sample preparation, we compared the protein precipitation (PP) method using methanol and acetonitrile and the liquid-liquid extraction (LLE) method using ethyl acetate, ethyl ether, methyl-tert-butyl ether, and methylene chloride to determine the most optimized sample preparation method. PP was not selected due to lower selectivity and recovery than LLE. According to the results of each method, the extraction using methylene chloride was better than other preparation methods in sensitivity, recovery, and intensity at a low concentration. For this reason, a simple LLE method using methylene chloride was adopted for the determination of CK41016.

### 2.2. Method Validation Result

Method validation was conducted according to the FDA Guidance for Industry: Bioanalytical Method Validation [26].

#### 2.2.1. Selectivity and Specificity

**Figure 2:**
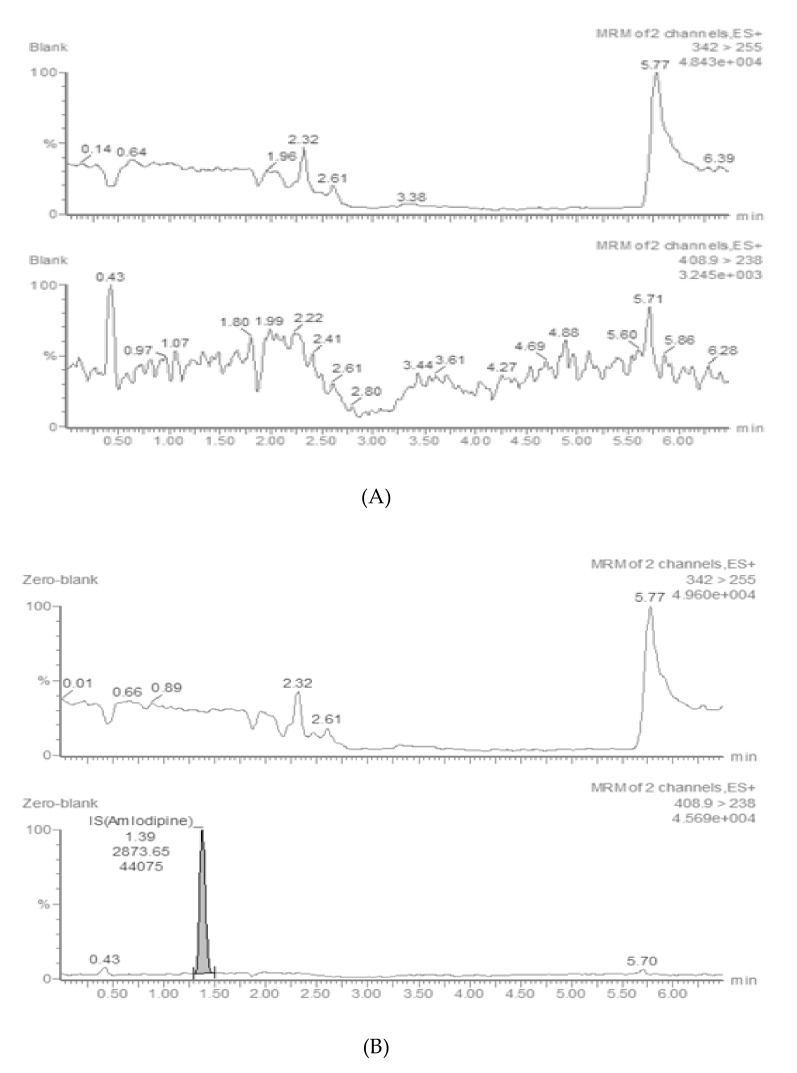

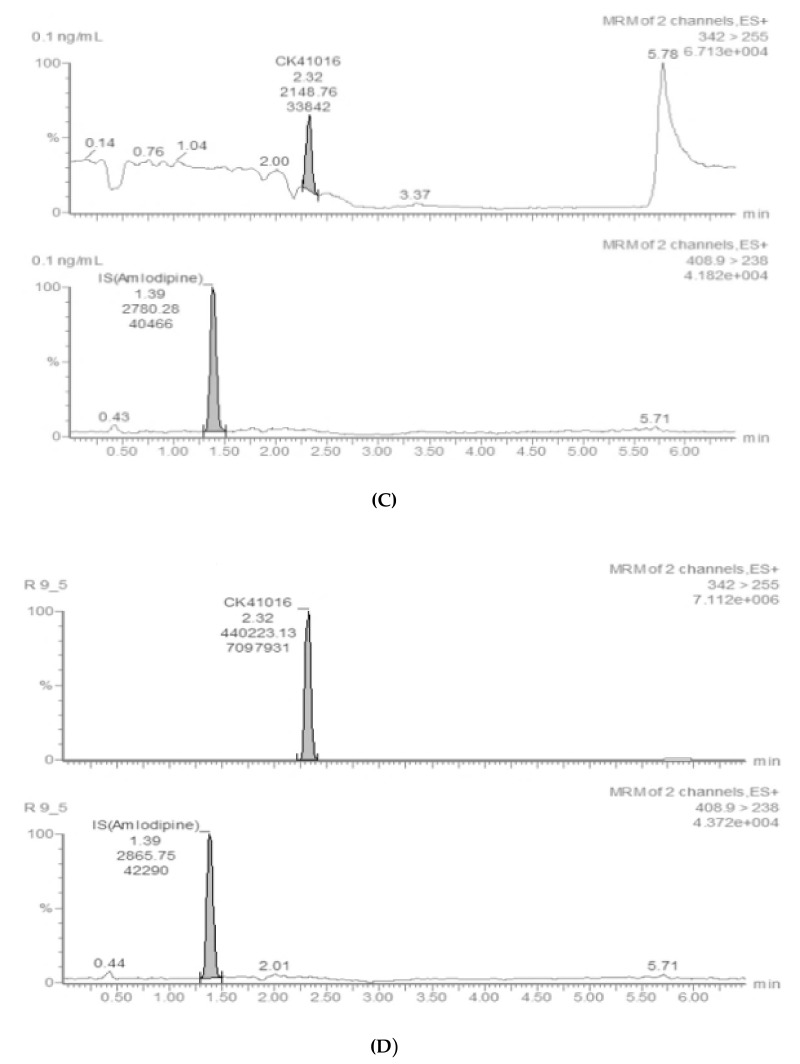
Representative multiple reaction monitoring (MRM) chromatograms of CK41016 in rat plasma. Blank rat plasma (**A**), zero-blank rat plasma spiked with IS (20 ng/mL) in extracted blank plasma (**B**), standard solution of CK41016 (0.1 ng/mL) and IS (**C**), and rat plasma taken at 5 min after eye drop administration of 75.21 μg/kg CK41016 (**D**).

The representative chromatograms of the blank plasma (A), spiked rat plasma with CK41016 at lower limit of quantification (LLOQ) of 0.1 ng/mL and the IS (20 ng/mL) in extracted blank plasma (B), standard solution of CK41016 (0.1 ng/mL) and IS (C), and rat plasma taken at 5 min after eye drop administration of 75.21 μg/kg CK41016 (D) are shown in Figure 2. As seen in the figure, the matrix effect in plasma and tissues of rats and rabbits did not exceed 15%, and no significant chromatographic interferences were observed with CK41016 and the IS at their retention times in drug-free plasma and tissues.

#### 2.2.2. Calibration Curves and LLOQ

The calibration curves of CK41016 showed a good linearity over the concentration range of 0.1–200 ng/mL with a correlation coefficient for all standard curves exceeding 0.992 at each batch (*n* = 5). The linear regression equations of the calibration curves for CK41016 are represented in Table 1, with *y* as the analyte peak-area ratio to the IS and *x* (ng/mL) as the analyte concentrations in plasma (or organ tissue). The LLOQ of CK41016 was 0.1 ng/mL. The UPLC-MS/MS analysis in this study provided a sufficient LLOQ for further PK study after the intravenous and eye drop administration of CK41016 in rats and rabbits.

#### 2.2.3. Precision and Accuracy

The intra-batch precision and accuracy at low, middle, and high quality control (QC) samples were in the ranges 2.52–5.02% and 95.33–103.93% (12.46% and 100.33% for the LLOQ), respectively. The inter-batch precision and accuracy at low, middle, and high QC samples were in the ranges 2.94–4.45% and 96.44–100.40% (1.80% and 102.11% for the LLOQ), respectively. These results indicate that all the values were within the acceptable range of ±15% for QC samples and ±20% for LLOQ, and this method showed satisfactory precision, accuracy, and reproducibility.

#### 2.2.4. Sensitivity

The LLOQ for this quantitative method is set as 0.1 ng/mL. The inter- and intra-batch results showed that LLOQ concentrations (*n* = 5) were within 100.33–102.11% of the theoretical value. Thus, it can be assured that the sensitivity for this quantitation method is appropriately set.

#### 2.2.5. Recovery and Matrix Effect

The extraction recovery of CK41016 from rat plasma was 88.16% ± 11.36%, whereas the recovery of the IS was 92.48% ± 6.50%. The mean matrix effects for CK41016 at low and high concentrations were 0.99–1.00, which indicated negligible suppression or enhancement.

#### 2.2.6. Stability

The QC samples (low and high concentration) of CK41016 were stable at room temperature (25 °C) for 24 h without any significant degradation, and the relative errors (REs) were within 12.19% for CK41016. Moreover, CK41016 was also considered stable after three freeze-thaw cycles in rat plasma (RE was less than 6.43%). The stock solutions of CK41016 and the IS stored at −20 °C in methanol were stable for a month, and the plasma samples stored at −80 °C were stable for three months, with a variation of <12.16%. All the results showed that CK41016 was stable under the different storage conditions.

#### 2.2.7. Incurred Sample Reanalysis

This assay was further evaluated using four rat samples in an incurred sample reanalysis (ISR). Two-third (67%) of the repeated sample results should be within 20% for small molecules and 30% for large molecules. The results of all the reanalyzed samples were within 10% [26].

All results were within the criteria of the FDA guidance. Thus, the developed bioanalytical method for determining CK41016 was successfully validated.

### 2.3. PK Study in Rats

**Figure 3:**
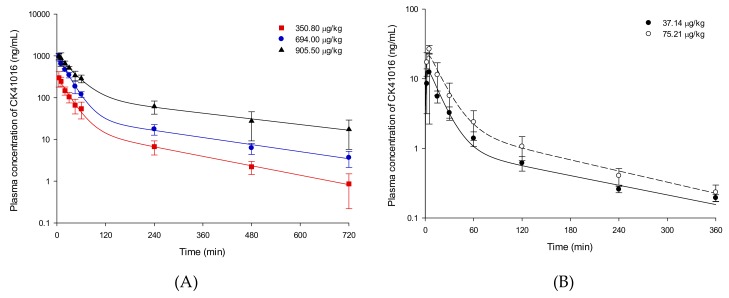
Plasma concentration-time profiles of CK41016 described by the two-compartment model after IV (**A**) or eye drop administration (**B**) in rats. Each value represents the mean ± SD (*n* = 3–6).

The validated method was applied to a PK study of CK41016 in rats after IV or eye drop administration. The plasma concentration–time curves of CK41016 after IV or eye drop administration are shown in Figure 3. The concentration–time profiles of CK41016 were adequately described by the two-compartment model without lag time using the WinNonlin^®^ software (version 8.1, Certara^TM^, Princeton, NJ, USA) program. The estimated results of PK parameters for CK41016 of each group are summarized in Table 2.

**Figure 4:**
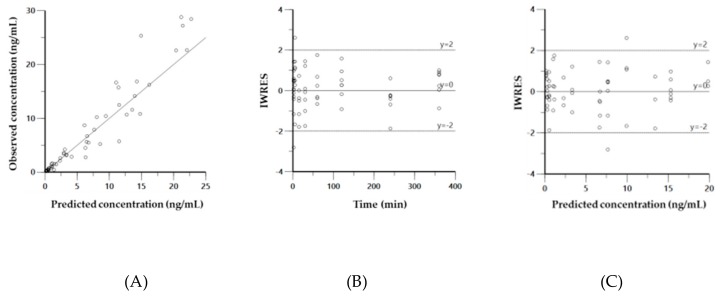
Diagnostic plots for the final pharmacokinetics (PK) model of CK41016 after eye drop administration in rats. Observed versus model-predicted concentration (**A**), individual weighted residual (IWRES) versus time (**B**), or model-predicted concentration (**C**).

Diagnostic plots for the final PK model of CK41016 after eye drop administration in rats, including the observed versus model-predicted concentration (A), individual weighted residual (IWRES) versus time (B), or model-predicted concentration (C) plots, are shown in Figure 4.

As seen from Table 2, the mean elimination half-life (t_1/2_) was approximately 206.83 min after IV administration. The AUC_0–∞,_ area under plasma concentration-time curve from time 0 to infinity, significantly increased in a dose disproportional manner from 12.58 μg·min/mL for 350.80 μg/kg to 76.25 μg·min/mL for 905.50 μg/kg. There was a significant (*p* < 0.05) difference in clearance (CL), which decreased nonlinearly from 27.88 to 11.88 mL/min/kg with the increasing dose. This result might be due to saturation of elimination by the enzyme-dependent clearance mechanism for the drug. After eye drop administration, the T_max_ was 4.30–4.40 min, suggesting that CK41016 was quickly absorbed into the body after administration of the eye drop.

Due to nonlinear PKs of CK41016 in rats, AUC_IV_, area under plasma concentration-time curve after IV administration, data of 350.80 μg/kg was used for the calculation of bioavailability (F). The F value of the CK41016 eye drop in rats was 31.53% for 37.14 μg/kg and 28.18% for 75.21 μg/kg.

**Figure 5:**
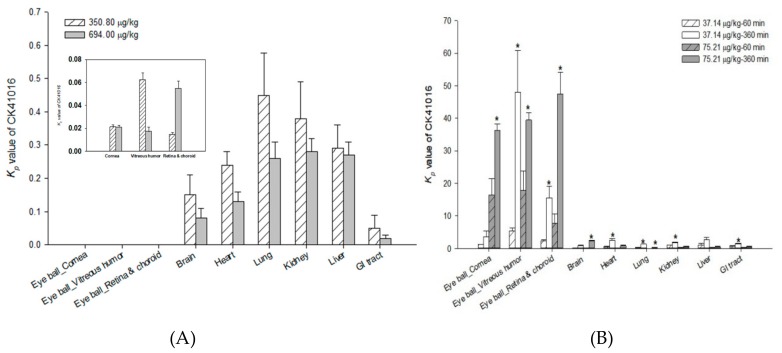
Mean tissue-to-plasma partition coefficient (Kp) values of CK41016 in rat tissues after IV (at 720 min) (**A**) or eye drop (60 and 360 min) administration (**B**) (mean ± SE, *n* = 5, * *p* < 0.05 between 60 and 360 min).

The tissue distribution of CK41016 was evaluated at 720 min after IV administration and at two timepoints (60 and 360 min) after eye drop administration in rats. The results are shown in Figure 5.

After eye drop administration in rats, CK41016 was rapidly distributed in tissues through systemic circulation. The tissue-to-plasma partition coefficient (Kp) value of CK41016 in almost all rat tissues has increased over timepoints; however, it was markedly higher in the eyeballs than that in any other tissues. This is probably due to the fact that the instilled dose of CK41016 was in direct contact with the eyes. CK41016 was mainly distributed in the vitreous humor of the eyeballs. Significant differences in Kp values at two timepoints were observed in the eyeball tissues. Since AMD is a disease affecting the posterior segment of the eye, the treatment target areas of this disease are retina and choroid. Therefore, the drug for the treatment of AMD should be delivered to the back of the eye. The Kp values in vitreous humor, retina, and choroid were over 1, suggesting that CK41016 might have been delivered and well-distributed in the target areas up to 360 min after eye drop administration.

### 2.4. PK Study in Rabbits

**Figure 6:**
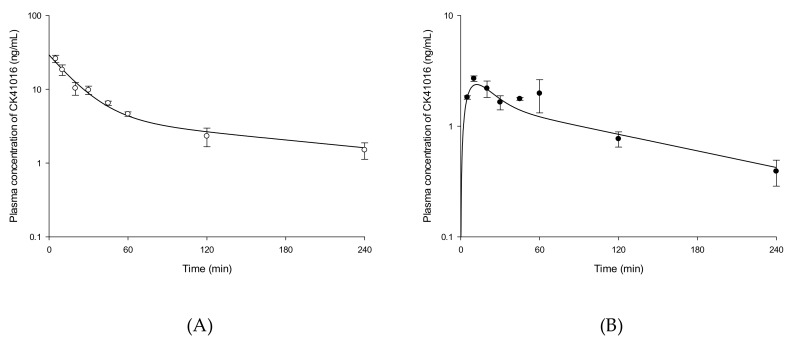
Plasma concentration-time profiles of CK41016 described by the two-compartment model after IV (**A**) or eye drop administration (**B**) in rabbits. Each value represents the mean ± SD (*n* = 5).

To investigate species differences, a PK study of CK41016 was evaluated in rabbits after IV or eye drop administration. Plasma concentration–time curves of CK41016 after IV or eye drop administration are shown in Figure 6. Similar to the results in rats, concentration–time profiles of CK41016 were adequately described by the two-compartment model without lag time using the WinNonlin^®^ software program. The estimated results of the PK parameters for CK41016 of each group are summarized in Table 3.

**Figure 7:**
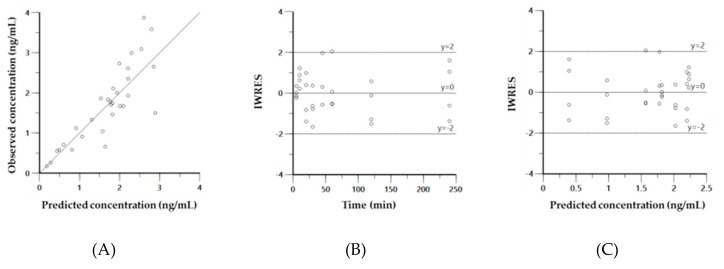
Diagnostic plots for the final PK model of CK41016 after eye drop administration in rabbits. Observed versus model-predicted concentration (**A**), individual weighted residual (IWRES) versus time (**B**), or model-predicted concentration (**C**).

Diagnostic plots for the final PK model of CK41016 after eye drop administration in rabbits, including the observed versus model-predicted concentration (A), individual weighted residual (IWRES) versus time (B) or model-predicted concentration (C) plots, are shown in Figure 7.

As shown in Table 3, the mean elimination half-life was approximately 104.38 or 88.45 min, and T_max_ was 15.39 min after eye drop administration. The absolute eye drop F of CK41016 in rabbits was 58.72%.

**Figure 8:**
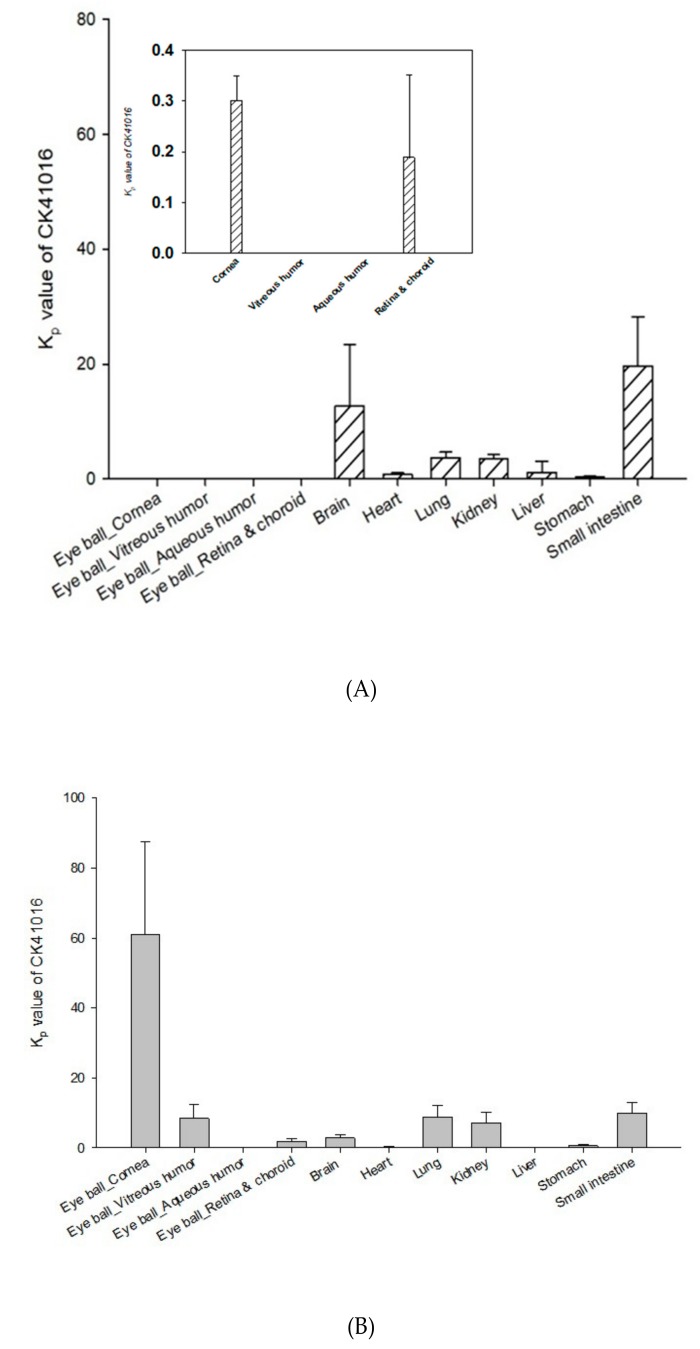
Mean Kp value of CK41016 in rabbit tissues at 240 min after IV (**A**) or eye drop administration (**B**) (mean ± SE, *n* = 5).

The tissue distribution of CK41016 was evaluated at 240 min after IV or eye drop administration in rabbits. Results are shown in Figure 8.

After eye drop administration in rabbits, CK41016 was distributed in major organs through systemic circulation, as well as the eyeball. Kp values of CK41016 in cornea, vitreous humor, retina, choroid, brain, lung, kidney, and small intestine were more than 1, indicating that CK41016 was well-distributed in these tissues at 240 min after eye drop administration. This suggests that CK41016 might have reached the posterior segment of the eyeball at 240 min after eye drop administration.

According to previous reports on the routes of drug delivery after eye drop administration [21,27], the ocular distribution of a drug is carried out via three major pathways after topical instillation: tear turnover, anterior (cornea/conjunctiva) region, and nasolacrimal drainage. Furthermore, there are four ways for a drug to reach the posterior of the eye. The drug will have to either (1) go through the cornea and aqueous chamber, penetrate the lens/iris to reach the vitreous before finally getting to the retina, (2) diffuse through the conjunctiva, sclera, and choroid to arrive at the retina, (3) go through horizontal diffusion from the cornea to the conjunctiva, or (4) go through the nasolacrimal drainage. The tissue distribution of CK41016 after eye drops in rats and rabbits shows that CK41016 was distributed to the cornea, vitreous humor, retina, and choroid but not to the aqueous humor. Based on the result, it can be hypothesized that the ocular penetration of CK41016 was carried out via a route similar to the pathway (2) from the report mentioned previously [27].

The F and tissue distribution in rabbits after eye drop administrations were different from those of rats. Although the highest distributed tissue was eyes in both rabbits and rats, the distribution trend within the eyeballs of rabbits was different from that of rats. After the eye drop administration in rats or rabbits, results showed that CK41016 reached the eyeballs in both species. However, less amounts of the drug were delivered to the retina of rabbits than in rats. This discrepancy might be due to the species differences. The corneal diameter and the anterior tissue volume of the rabbits are 2- and 18-fold larger than that of the rats, respectively [28]. This could explain why more CK41016 was distributed in the cornea of the anterior segment but less in the posterior tissue in rabbits than in rats.

### 2.5. Transport Assay of CK41016

#### 2.5.1. Cytotoxicity Assay of CK41016

**Figure 9:**
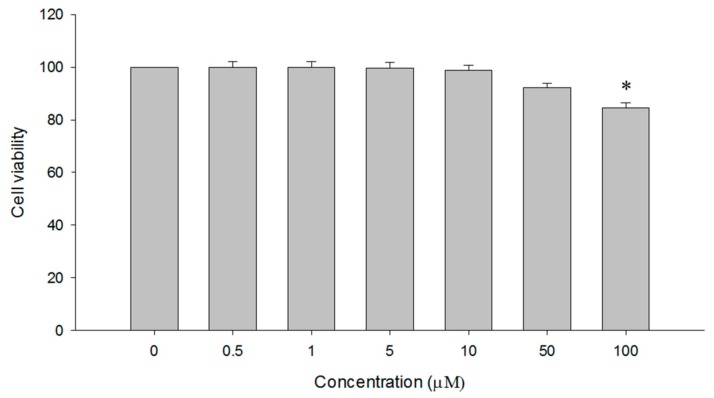
Cytotoxicity of CK41016 in Caco-2 cells (*n* = 6, mean ± SD, * *p* < 0.05 between control).

Cytotoxicity of CK41016 at different concentrations to Caco-2 cells was evaluated using a CCK-8 kit. A CCK-8 assay is a sensitive colorimetric assay to determine the number of viable cells in cell proliferation and cytotoxicity assays. Figure 9 shows the cell viability of Caco-2 cells after treatment with 0 (control), 0.5, 1, 5, 10, 50, and 100 µM of CK41016 for 24 h. Results of the CCK-8 assay showed that treatment with CK41016 at 0.5, 1, 5, 10, or 50 μM had no cytotoxicity to Caco-2 cells. Based on these results, the treatment concentration of CK41016 for the bidirectional transport assay was chosen.

#### 2.5.2. Efflux Transport Assay of CK41016

Based on the results of cytotoxicity assay, the bidirectional membrane transport of CK41016 at concentrations of 10, 20, 30, and 50 µM in Caco-2 cell monolayers was evaluated. CK41016 was stable after 2 h of incubation in the transport medium at 37 °C. As shown in Table 4, the permeability of CK4101 increased in a concentration-dependent manner. According to FDA guidance [29], any investigational drug with an efflux ratio (ER) greater than two is considered a potential substrate of active efflux transporters. ER of CK41016 was calculated to be 0.73 to 1.21 (Table 4), indicating that CK41016 was not a substrate of active efflux transporters. It also suggested that no significant efflux was involved in the membrane transport of CK41016. Permeability of CK41016 increased with concentration dependently. These results indicated that CK41016 permeated the membrane through passive diffusion.

There are many difficulties in delivering a drug to the posterior of the eye, since such delivery is limited by the blood-retina barrier, efflux transporter, and others [21,25]. The experimental results suggest that CK41016 was not a substrate of the efflux transporter, such as P-gp, and CK41016 was distributed in the retina (Figure 5B and Figure 8B). CK 41016 not being the substrate of the efflux transporter could be one of the reasons why it was able to be delivered to the posterior segment of the eye, the therapeutic target for dry AMD. These results could support CK41016 eye drops as a potential dry AMD treatment.

## 3. Materials and Methods

### 3.1. Chemicals and Reagents

**Figure 10:**
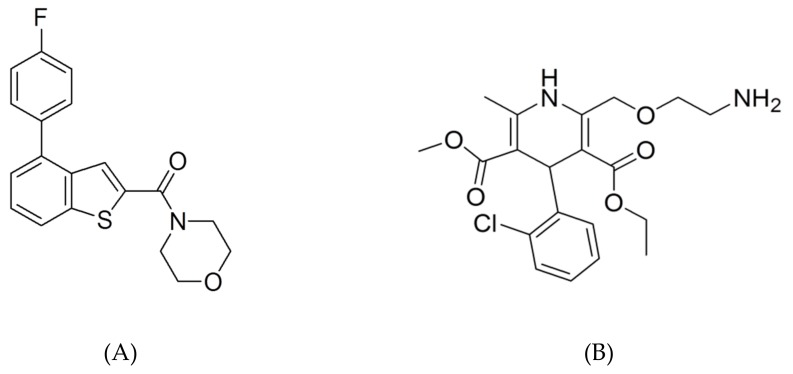
Chemical structures of CK41016 (**A**) and amlodipine (IS) (**B**).

Chemical structures of CK41016 (purity >99.5%) and amlodipine (purity >99.9%) as IS are shown in Figure 10. CK41016 was provided by the Kukje Pharmaceutical Co. (Gyeonggi-do, Korea), and the IS was purchased from Sigma-Aldrich (St. Louis, MO, USA). Methanol, acetonitrile, tetrahydrofuran, and methylene chloride were purchased from J.T. Baker (Phillipsburg, NJ, USA). Formic acid was purchased from Sigma-Aldrich (St. Louis, MO, USA). Distilled water (18.2 MΩ) was obtained with an Elga Purelab Option-Q System (Elga Labwater, Marlow, UK). It was used throughout this study. All other chemicals were of HPLC or analytical grade.

### 3.2. Quantification of CK41016 in Biological Samples

The quantitation of CK41016 in biological samples (such as plasma and tissues of rats and rabbits and the transport medium) was performed with a newly developed bioanalytical method using an Acquity UPLC^®^ System coupled with a mass spectrometer (Xevo TQ-S, Waters Corp., Milford, MA, USA). Chromatography was performed with a Kinetex biphenyl column (2.1 mm × 50 mm, 1.7 µm particle size, Phenomenex, Torrance, CA, USA) at a temperature of 25 ± 5 °C. The mobile phase consisted of 0.1% formic acid in water:tetrahydrofuran (9:1, *v/v*) and 0.1% formic acid in acetonitrile:tetrahydrofuran (9:1, *v/v*), with a gradient condition at a flow rate of 0.3 mL/min. The elution program was as follows: 0–0.5 min (25% B), 0.5–3.5 min (25–90% B), 3.5–5.0 min (90% B), 5.0–5.1 min (90–25% B), and 5.1–6.5 min (25% B). The mass spectrometer was operated in a multiple-reaction monitoring (MRM) mode with an electrospray ionization interface in the positive ion mode. Mass transitions were respectively *m/z* 342.1→255.1 for CK41016 and *m/z* 409.1→238.2 for the IS. Optimized parameters were accomplished as follows: capillary voltage, 3.3 kV; ion source temperature, 150 °C; desolvation temperature, 350 °C; flow rate of cone gas, 150 L/h; flow rate of desolation gas, 600 L/h; and pressure of argon gas, 4.2 × 10^−3^ mbar. The optimal collision energy was 20 eV for CK41016 and 12 eV for IS. The cone voltage was 38 V for CK41016 and 28 V for IS. Data acquisition and analysis were performed with Masslynx 4.1 software (Waters Corp., Milford, MA, USA).

CK41016 was extracted from plasma, tissues, and transport medium samples with a simple LLE method. Tissue samples were homogenized (Ultra-Turrax^®^, IKA^®^ T10 basic, Staufen, Germany) in five volumes of distilled water. To 50 µL of each sample, 10 µL of IS (amlodipine 20 ng/mL in 50% acetonitrile) was added using a repeating pipette. Then 900 µL of methylene chloride was added and stirred for 3 min before centrifuging at 15,000× *g* for 5 min at room temperature. After centrifugation, 800 µL of the organic layer was transferred to a clean micro tube and evaporated at room temperature using a nitrogen evaporator. The residue was reconstituted in 100 µL of 0.1% formic acid in 50% acetonitrile, stirred for 1 min, and centrifuged at 15,000× *g* for 5 min. Then 5 µL of the supernatant was injected to a UPLC-MS/MS for analysis. The developed bioanalytical method was validated according to the FDA Guidance for Industry: Bioanalytical Method Validation [26].

### 3.3. Animals and PK Study in Rats

Normal male Sprague-Dawley rats (8–12 weeks, 250~270 g) were obtained from Orient Bio Inc. (Gyeonggi-do, Korea). All animals were separately kept in metabolic cages in an air-conditioned room with a controlled temperature of 23 ± 2 °C, a relative humidity of 50% ± 10%, and 12 h of a light/dark cycle. Rats were used for experiments after one week of acclimation. All animal experiments were approved by the Institutional Animal Care and Use Committee (IACUC, No. IACUC 180114) in CHA Laboratory Animal Research Center (Seongnam, South Korea).

According to administration doses or routes, male rats were randomly divided into five groups. In three groups, CK41016 was administered intravenously by a tail vein at 350.80, 694.00, or 905.50 μg/kg (*n* = 3/group). The other two groups were instilled with one drop (15 μL) of CK41016 eye drop into both eyes, which was equivalent to 37.14 or 75.21 μg/kg (*n* = 6/group). Blood samples were collected before administration and at predetermined times after administration (Table 5). Approximately 250 μL of blood was collected via the jugular vein periodically into heparinized tubes. During our experiment, the rats were allowed to move freely in their metabolic cage, and blood samples were collected without rat fixation and anesthesia. Rats were also provided hydration via zonde to compensate for their blood loss. All collected blood was immediately centrifuged at 3000× *g* for 15 min, and samples were stored at −80 °C until analysis. At predetermined times after IV or eye drop administration, tissues were sampled after being sacrificed. Tissue samples of eyeballs (cornea, vitreous humor, retina, and choroid); liver; kidney; heart; lung; brain; and GI tract were collected, weighed, and stored at −80 °C until analysis. PK parameters were calculated by compartmental analysis using a WinNonlin^®^ software version 8.1 (Certara™, Princeton, NJ, USA). Differences of PK parameters between groups were tested by one-way analysis of variance (ANOVA). The Mann-Whitney U-test was used to evaluate the difference of the Kp value of each tissue at two time points. Statistical tests were performed with R software (R Core Team (2018), R: A language and environment for statistical computing. R Foundation for Statistical Computing, Vienna, Austria. (URL https://www.R-project.org/). Significant difference was considered at *p* < 0.05.

The absolute eye drop bioavailability (F) of CK41016 was calculated according to the following equation:

(1) $$F\;\left(\%\right)=\left(\frac{AUC_{eye\;drop}/Dose_{eye\;drop}}{AUC_{IV}/Dose_{IV}}\right)\times 100$$

where area under plasma concentration-time curve from time 0 to infinity (AUC_eye_) _drop_ and area under plasma concentration-time curve after IV administration (AUC_IV_) were the AUC values after eye drop and IV administration of CK41016, and Dose_eye drop_ and Dose_IV_ were actual doses via eye and IV administration, respectively.

### 3.4. Animals and PK Study in Rabbits

Male New Zealand White rabbits were obtained from Koatech Inc. (Gyeonggi-do, Korea) to evaluate PKs in rabbits. They were kept in an air-conditioned room maintained with a 12 h light-dark cycle, a controlled ambient temperature (23 ± 2 °C), and a humidity of 50% ± 10%. This study was approved by the Institutional Animal Care and Use Committee (IACUC, No. IACUC 190032) of the CHA Laboratory Animal Research Center (Seongnam, South Korea).

Depending on administration route, CK41016 (66.00 μg/kg) was intravenously administered by ear vein or 1 drop (50 μL) of CK41016 was instilled into each eye of the rabbits, which was equivalent to 20.98 μg/kg (*n* = 5/group). Blood samples were collected before administration and at 5, 10, 20, 30, 45, 60, 120, and 240 min after the drug administration (Table 6). Approximately 1 mL of blood was collected via ear artery into heparinized tubes with an IV catheter (BD, Franklin Lakes, New Jersey, USA). Blood samples were immediately centrifuged at 3000× *g* for 15 min and then stored at −80 °C until analysis. After the last blood sample was obtained, rabbits administered with IV or eye drop were sacrificed, and tissues such as eyeballs, liver, kidney, heart, lung, brain, stomach, and small intestine were collected. Eyeballs were additionally separated into cornea, vitreous humor, aqueous humor, retina, and choroid. All tissue samples were stored at −80 °C until analysis after obtaining their weights. The PK parameters of CK41016 were calculated by compartmental analysis using the WinNonlin^®^ software version 8.1 (Certara™, Princeton, NJ, USA).

### 3.5. Efflux Transport Assay

#### 3.5.1. Cell Culture

Caco-2 cells were obtained from the American Type Culture Collection (Manassas, VA, USA) and grown in Dulbecco’s modified Eagle medium (DMEM) supplemented with 10% fetal bovine serum (Corning, NY, USA) and 1% penicillin streptomycin (Corning, NY, USA) in an atmosphere of 5% CO_2_ at 37 °C in a CO_2_ incubator (Heraeus BB15, Thermo Fisher Scientific Inc., MA, USA). The medium was changed every other day and harvested every 3–5 days with trypsin-EDTA. For transport study, Caco-2 cells with passage number 51 were seeded into 12-well Transwell plates (1.12 cm^2^ in surface, 0.4 μm in pore size, 12 mm in diameter; Corning Costar Corporation, MA, USA) at a density of 1.9 × 10^7^ cells/well. These cells were cultured for 21 days. The medium was replaced every other day. The integrity and transporter ability of cell monolayers were evaluated by measuring transepithelial electrical resistance (TEER) with an epithelial voltohmmeter (EVOM2, World Precision Instrument, Sarasota, FL, USA) before transport experiments. Caco-2 cell monolayers with TEER values exceeding 500 Ω cm^2^ were used in the transport study.

#### 3.5.2. In Vitro Cytotoxicity Assay

Cytotoxicity of CK41016 to Caco-2 cells was evaluated using a Cell Counting Kit-8 (CCK-8) (Dojindo Molecular Technologies, Inc., Rockville, MD, USA). First, Caco-2 cells were seeded into 96-well plates at a density of 1.0 × 10^4^ cells/well and preincubated at 37 °C for 24 h with an atmosphere of 5% CO_2_. Then, 10 µL of CK41016 at various concentrations was added into each well, followed by incubation for 24 h in the incubator at same condition. A 10 µL of CCK-8 reagent was added to each well of the 96-well plate. After incubation at 37 °C with 5% CO_2_ for 4 h, absorbance of each well was measured at 450 nm using a 96-well plate reader (SpectraMax i3x, Molecular Devices, LLC, San Jose, CA, USA). Cell viability (%) was calculated based on the measured value relative to the absorbance of cells exposed to the negative control. All data are shown as mean ± standard deviation (*n* = 6). Statistical comparisons were made with Student’s *t*-test. *P*-values of less than 0.05 were considered statistically significant.

#### 3.5.3. Transport Assay

A bidirectional transport assay was conducted based on related previous reports and FDA guidance [27]. Before performing the transport assay, cells monolayers were washed twice using a prewarmed (37 °C) transport medium (Hank’s balanced salt solution with 10 mM HEPES, pH7.4). Cells were equilibrated at 37 °C in an atmosphere of 5% CO_2_ for 30 min with the transport medium. Then, 10 mM stock solution of CK41016 was prepared in DMSO and diluted with transport medium to 10, 20, 30, and 50 μM.

The transport assay was conducted by adding each concentration of CK41016 to either the apical (AP, 0.5 mL) or basolateral side (BL, 1.5 mL). The receiver side was added the corresponding same volume of the transport medium. Plates were shaken gently for 2 h at 37 °C on a plate shaker. After 200 μL samples were taken from the basolateral (for AP-BL transport) or apical (for BL-AP transport) chamber at 15, 30, 45, 60, 90, and 120 min, the same volume of fresh transport medium was added as replacement. After taking the last samples, samples were also collected from the apical (for AP-BL transport) or basolateral (for BL-AP transport) chamber to analyze mass balance. Each sample was immediately frozen and stored at −80 °C until analysis with established UPLC-MS/MS method. The apparent permeability coefficients (P_app_) were calculated as shown below:

(2) $$P_{\mathrm{app}}=(\mathrm{dQ}/\mathrm{dt})/\left(A\times C_{0}\right)$$

where dQ/dt (μmol/s) was the cumulative rate transported of CK41016 on the receiver side, A (cm^2^) was the membrane surface area, and C_0_ (μM) was the initial concentration in the donor compartment.

The efflux ratio was calculated with the following equation:

(3) $$\mathrm{Efflux}\;\mathrm{ratio}=P_{app}\left(BL\to AP\right)/P_{app}\left(AP\to BL\right)$$

where P_app_ (BL→AP) was the P_app_ of the basolateral side to the apical side, and P_app_(AP→BL) was the P_app_ of the apical side to the basolateral side.

## 4. Conclusions

In this study, a selective, sensitive, and reliable UPLC-MS/MS method was developed and validated for the quantification of CK41016. The PK model and tissue distribution of CK41016 were successfully evaluated after IV or eye drop administrations to rats and rabbits. The efflux transport mechanism of CK41016 was also investigated using a Caco-2 cell monolayer model. The discrepancy in PK parameters and tissue distribution trends between rats and rabbits might be due to species differences, and these features should be considered in clinical trials. In addition, metabolic mechanisms of CK41016 that might be associated with the nonlinear PK of the drug should be further investigated via metabolic assays. Additionally, the causes for differences in PKs and tissue distribution among species need to be investigated to predict PKs of CK41016 in humans in the future. Overall, the results of this study suggest that CK41016 eye drops might be a potential new drug candidate for dry AMD treatment.

## Figures and Tables

**Table 1:** Calibration curves of CK41016 in plasma and tissues of rats and rabbits.

Matrix		Calibration Curve (Rat)	Calibration Curve (Rabbit)
**Plasma**		y = (0.21 ± 0.01)x + (0.01 ± 0.01)	y = (1.00 ± 0.06)x + (0.22 ± 0.06)
**Eye ball**	Cornea	y = (0.31 ± 0.01)x + (0.14 ± 0.004)	y = (0.98 ± 0.12)x + (0.12 ± 0.05)
	Vitreous humor	y = (0.32 ± 0.03)x + (0.13 ± 0.02)	y = (0.95 ± 0.11)x + (0.10 ± 0.02)
	Retina & choroid	y = (0.16 ± 0.01)x + (0.04 ± 0.01)	y = (1.03 ± 0.14)x + (0.07 ± 0.05)
	Aqueous humor ^a^	−	y = (1.14 ± 0.07)x + (0.07 ± 0.20)
**Heart**		y = (0.17 ± 0.01)x + (0.01 ± 0.01)	y = (0.80 ± 0.09)x + (0.08 ± 0.08)
**Liver**		y = (0.12 ± 0.01)x + (0.04 ± 0.04)	y = (0.99 ± 0.10)x + (0.28 ± 0.17)
**Brain**		y = (0.12 ± 0.004)x − (0.01 ± 0.04)	y = (0.98 ± 0.10)x + (0.05 ± 0.05)
**Lung**		y = (0.17 ± 0.01)x + (0.01 ± 0.04)	y = (0.90 ± 0.12)x + (0.13 ± 0.03)
**Kidney**		y = (0.15 ± 0.01)x + (0.02 ± 0.01)	y = (0.86 ± 0.12)x + (0.16 ± 0.14)
**GI tract** ^b^	Stomach	y = (0.13 ± 0.01)x + (0.01 ± 0.04)	y = (0.96 ± 0.11)x + (0.12 ± 0.12)
	Small intestine		y = (0.69 ± 0.06)x + (0.06 ± 0.10)

^a^ The matrix of the rats did not include aqueous humor. ^b^ GI tract of rabbits were classified into stomach and small intestine.

**Table 2:** Pharmacokinetics (PK) parameters of CK41016 following IV or eye drop administration in rats (mean ± SE, *n* = 3–6).

Parameter	IV			Eye Drop		
	350.80 μg/kg	694.00 μg/kg	905.50 μg/kg		37.14 μg/kg	75.21 μg/kg
CL (mL/min/kg) ^a,^ *	27.88 ± 1.18	19.28 ± 0.75	11.88 ± 0.31		88.69 ± 3.36	93.68 ± 5.06
CL_D_ (mL/min/kg) ^a^	10.03 ± 1.35	6.71 ± 0.73	9.54 ± 0.81		57.60 ± 6.56	46.48 ± 2.92
V (L/kg) ^a^	1.07 ± 0.10	0.65 ± 0.06	0.82 ± 0.05		1.92 ± 0.22	1.58 ± 0.75
V_2_ (L/kg) ^a^	1.63 ± 0.16	1.48 ± 0.16	1.85 ± 0.18		7.20 ± 1.28	5.16 ± 0.92
C_max_ (μg /mL) ^b^	0.33 ± 0.03 *	1.06 ± 0.09 *	1.09 ± 0.06		0.01 ± 0.004 *	0.02 ± 0.002 *
T_max_ (min)	−	−	−		4.30 ± 1.50	4.40 ± 1.00
AUC_0–∞_ (μg·min/mL) *	12.58 ± 0.53	35.99 ± 1.40	76.25 ± 2.00		0.42 ± 0.05	0.76 ± 0.04
t_1/2_ (min)	160.70 ± 13.09	213.18 ± 21.62	265.68 ± 23.08		129.28 ± 32.11	112.04 ± 9.44
F (%) ^†^	−	−	−		31.53	28.18

^a^ The value refers to the clearance (CL/F and CL_D_/F) and volume of distribution (V/F and V_2_/F) after eye drop administration. ^b^ The concentration is the C_0_ (ng/mL) after IV administration. ^*^ *p* < 0.05 are significant differences according to dose within IV or eye drop administration groups. ^†^ Area under plasma concentration-time curve after IV administration (AUC_IV_) data of 350.80 μg/kg was used for the calculation of bioavailability (F) (%). AUC_0–∞,_ area under plasma concentration-time curve from time 0 to infinity.

**Table 3:** PK parameters of CK41016 following IV or eye drop administration in rabbits (Mean ± SE, *n* = 5).

Parameter	IV	Eye Drop
	66.00 µg/kg	20.98 µg/kg
CL (mL/min/kg) ^b^	43.90 ± 6.36	77.26 ± 13.18
CL_D_ (mL/min/kg) ^b^	51.70 ± 9.54	182.78 ± 31.18
V(L/kg) ^b^	2.35 ± 0.29	7.03 ± 2.87
V_2_ (L/kg) ^b^	5.74 ± 0.38	18.27 ± 9.90
C_max_ (ng/mL) ^a^	28.14 ± 3.47	2.52 ± 0.41
T_max_ (min)	−	15.39 ± 4.88
AUC_0–∞_ (μg·min/mL)	1.50 ± 0.22	0.28 ± 0.04
t_1/2_ (min)	104.38 ± 57.41	88.45 ± 32.13
F (%)	−	58.72

^a^ The concentration is the C_0_ (ng/mL) after IV administration. ^b^ The value refers to the clearance (CL/F and CL_D_/F) and volume of distribution (V/F and V_2_/F) after eye drop administration.

**Table 4:** Permeability and efflux ratio of CK41016 in Caco-2 cell monolayers.

CK41016 (μM)	Caco-2 P_app_ (×10^−6^ cm/sec)		Efflux Ratio
	AP→BL	BL→AP	
10	11.52 ± 0.55	13.88 ± 0.35	1.21
20	27.50 ± 2.47	27.59 ± 0.66	1.01
30	32.96 ± 1.47	30.11 ± 1.97	0.92
50	49.05 ± 3.53	35.98 ± 2.55	0.73

**Table 5:** Experimental design of PK studies in rats.

Group	Route	Dose (μg/kg)	Sampling Time (min)
1	IV	350.80	5, 10, 20, 30, 45, 60, 240, 480, 720 ^a^
2		694.00	
3		905.50	
4	Eye drop	37.14	2, 5, 15, 30, 60^*^, 120, 240, 360 ^b^
5		75.21	

^a^ After IV administration, tissues were sampled at 720 min (350.80 and 694.00 μg/kg). ^b^ After eye drop administration, tissues were sampled at 60 and 360 min.

**Table 6:** Experimental design of PK studies in rabbits.

Group	Route	Dose (μg/kg)	Sampling Time (min)
1	IV	66.00	5, 10, 20, 30, 45, 60, 120, 240
2	Eye drop	20.98	
